# Telomere shortening leads to an acceleration of synucleinopathy and impaired microglia response in a genetic mouse model

**DOI:** 10.1186/s40478-016-0364-x

**Published:** 2016-08-22

**Authors:** Annika Scheffold, Inge R. Holtman, Sandra Dieni, Nieske Brouwer, Sarah-Fee Katz, Billy Michael Chelliah Jebaraj, Philipp J. Kahle, Bastian Hengerer, André Lechel, Stephan Stilgenbauer, Erik W. G. M. Boddeke, Bart J. L. Eggen, Karl-Lenhard Rudolph, Knut Biber

**Affiliations:** 1 Department of Internal Medicine III, University of Ulm, Albert-Einstein-Allee 23, 89081 Ulm, Germany; 2 Department of Neuroscience, Section Medical Physiology, University of Groningen, University Medical Center Groningen, 9713 AV Groningen, The Netherlands; 3 Department for Psychiatry and Psychotherapy, Molecular Psychiatry, Freiburg University Hospital, Hauptstrasse 5, 79104 Freiburg, Germany; 4 Department of Internal Medicine I, University of Ulm, Albert-Einstein-Allee 23, 89081 Ulm, Germany; 5 Center of Neurology and Hertie-Institute for Clinical Brain Research, University of Tübingen, Tübingen, Germany; 6 Boehringer Ingelheim Pharma GmbH & Co. KG, Biberach an der Riss, Germany; 7 Leibniz Institute for Age Research, Beutenbergstr. 11, 07745 Jena, Germany

**Keywords:** Parkinson’s disease, α*-*synuclein, Telomeres, Microglia

## Abstract

Parkinson’s disease is one of the most common neurodegenerative disorders of the elderly and ageing hence described to be a major risk factor. Telomere shortening as a result of the inability to fully replicate the ends of linear chromosomes is one of the hallmarks of ageing. The role of telomere dysfunction in neurological diseases and the ageing brain is not clarified and there is an ongoing discussion whether telomere shortening is linked to Parkinson’s disease. Here we studied a mouse model of Parkinson’s disease (Thy-1 [A30P] *α-*synuclein transgenic mouse model) in the background of telomere shortening (Terc knockout mouse model). *α-s*ynuclein transgenic mice with short telomeres (αSYN^tg/tg^ G3Terc^-/-^) developed an accelerated disease with significantly decreased survival. This accelerated phenotype of mice with short telomeres was characterized by a declined motor performance and an increased formation of α-synuclein aggregates. Immunohistochemical analysis and mRNA expression studies revealed that the disease end-stage brain stem microglia showed an impaired response in αSYN^tg/tg^ G3Terc^-/-^ microglia animals. These results provide the first experimental data that telomere shortening accelerates *α-*synuclein pathology that is linked to limited microglia function in the brainstem.

**Electronic supplementary material:**

The online version of this article (doi:10.1186/s40478-016-0364-x) contains supplementary material, which is available to authorized users.

## Introduction

Parkinson’s disease (PD) is a neurodegenerative disease in the elderly with an average age of onset of 60 years, with 8-18/100,000 newly diagnosed patients every year [1]. The main characteristic of PD is the death of dopaminergic neurons in the substantia nigra pars compacta (SN) in the brain of PD patients. Several missense mutations and genomic multiplications of the α-synuclein gene (*SCNA*) are described to cause the autosomal dominant hereditary PD and SCNA polymorphisms are major genetic risk factors for PD [2].

Aggregated α-synuclein protein forms immuno-reactive inclusions which incorporate in typical Lewy bodies and Lewy neurites [3] and induce neuroinflammation in humans [4]. As a pathological hallmark of PD, Lewy bodies were for a long time thought to be the key component of the neuronal cell death and a pathological hallmark of PD, however the process of soluble monomers into insoluble α-synuclein aggregates reached increasing attention as the disease causative step [5–7]. Prefibrilliar forms induce cell death in vitro [8] but the causal mechanism of α-synuclein oligomerization has not yet been identified.

Microglia are of crucial importance in brain pathology, and these cells are currently in focus as potential targets for improved therapies [9]. It has now been established in mice that microglia arise from the yolk sac erythromyeloid precursors that invade the brain rudiment around embryonic day 9.5 in the mouse [10–12]. Based on cell morphology it was originally assumed that the ramified (branched) microglia in the healthy brain are inactive or resting and that microglia under pathological conditions acquire an amoeboid morphology described as “activated microglia”. Since ramified microglia are by no means resting cells [13–15] the simple concept of microglia “activation” in disease is misleading. Instead of becoming “activated” microglia undergo a disease-specific phenotype shift, which might be associated with tissue repair or with enhanced pro-inflammatory activity, thus contributing to the disease [16–23]. Thus, understanding how microglia function in diseases is regulated and addressing the contribution of microglia to brain diseases is of pivotal importance.

Parkinson’s disease is highly connected to neuroinflammatory changes with the presence of amoeboid or reactive microglia both in patients and various Parkinson’s disease models [24]. However, the precise role of microglia in the disease is still under debate as potential detrimental as well as protective properties of these cells have been published [25]. For example microglia may also restrict the disease by gluthatione peroxidase expression which is protective against Lewy body formation [26].

Since Parkinson’s disease is a prominent disease of the elderly, ageing is supposed to be a major risk factor [27–29]. Microglia are affected in the ageing brain and nevertheless it is still not explained, if ageing related changes or chronic disease prime glial cells to induce neurotoxicity or whether microglia ageing impairs their function which in turn may promote neurodegeneration [30]. Several pathways and key genes, as well as telomere shortening which occurs with cell replication are involved in the process of ageing [31]. Telomere length is therefore considered to be an indicator of biological ageing. Intensive studies have confirmed, that chronic diseases lead to accelerated shortening of the chromosomal ends [32, 33]. Telomere shortening in microglia cells of ageing rats has already been described in vitro as well as in vivo [34, 35]. Telomere shortening of microglia leads to cellular senescence and is associated with amyloid dementia [36].

A possible link between telomere shortening in peripheral leukocytes and Parkinson’s disease has been addressed with inconclusive results. In some studies telomere shortage was linked to accelerated disease, whereas in other reports there was no clear correlation between telomere length and disease progression [37–40].

In order to further understand the potential role of telomere shortening in Parkinson’s disease pathology we crossed the α-synuclein transgenic Parkinson mouse model Thy-1 [A30P] with the Terc knockout mice, a telomere erosion-based ageing mouse model.

## Materials and methods

### Mouse models

The αSYN transgenic mice used here express the human mutant [A30P] α*-*synuclein under control of the neuron-specific Thy-1 promoter [41, 42]. Genotyping of mice was performed as previously described [42, 43].

Terc knockout mice carry a homozygous deletion of the telomerase RNA subunit Terc. Thereby telomerase activity is lost [44]. Like the αSYN transgenic mice, Terc knockout mice are in the same C57/BL6/J strain, minimizing genetic background influences.

For generating homozygous αSYN^tg/tg^ G3Terc^-/-^ mice, heterozygous αSYN^tg/wt^ Terc^+/-^ mice were crossed to get the first generation of Terc knockout (αSYN^tg/tg^ Terc^-/-^ G1). Crossing of the homozygous 1st Terc^-/-^ generation was performed to obtain the 2nd generation, and crossing of the 2nd generation results in the 3rd generation of Terc knockout mice.

All mice were maintained and bred at the animal facility of Ulm University (Tierforschungszentrum Ulm). The mice were maintained in a pathogen-free environment (SPF IVC barrier) with a 14/10 h day and night rhythm. Food and drinking water were available *ad libitum*. Temperature and humidity were controlled at 23 °C.

All animal experiments were performed according to protocols approved by the state government of Baden Württemberg, following the animal welfare guidelines.

### Beamwalk

Beam walking is a well established test to monitor motor coordination and balance of rodents. Mice were trained for three consecutive days to cross a squared, wooden beam to reach an enclosed platform. The latency and the footslips to traverse the beam were measured. Mice were trained for four consecutive days on the squared 28 mm beam. Each mouse completed three trainings per day. For final testing, three rounds on four different beams were performed. The squared beams had a side length of 5, 12, and 28 mm, the round beams had a diameter of 11, 17, and 28 mm. Parameters such as the latency and footslips and the number of falls were recorded and analyzed as previously described [45].

### Exploratory behavior

Exploratory behavior of the transgenic mice was analyzed using the Viewer^3^ system (Biobserve) [46]. This system contains an activity box, which is subdivided into three different zones, the border, the intermediate and the center zone. During the experiment, several parameters such as the activity, duration in different zones and the totally walked distance were recorded. Mice were put into the exploratory fields for 10 min. After each run, boxes were cleaned with ddH_2_O and 70 % Ethanol to remove pheromones.

### Transcardial perfusion and brain tissue collection

αSYN transgenic symptomatic mice develop a phenotype with severe motoric imbalances and dysfunctions of brainstem and cerebellum [42]. Dead criteria were defined when the mice start to develop motoric imbalances and their walk was disturbed. To collect brain tissue, mice were transcardially perfused. Briefly, mice were anaesthetized with Ketamin/Xylazin. After loss of reflexes, the heart cavity of the mouse was opened, the mouse has been perfused for 3 min using ice-cold PBS to flush and remove blood from the vessels. The brain was dissected and cut into the hemispheres, one hemisphere was snap-frozen in liquid nitrogen and stored at −80 °C, the other half was fixed in 4 % PFA overnight and embedded in paraffin the next day.

### α-Synuclein staining on proteinase K digested paraffin embedded tissue blots (PK-PET Blot)

The Proteinase K digested paraffin-embedded tissue (PK-PET) blot was performed as described previously [47], with minor modifications in the protocol. A 0.45 μm PVDF membrane (Serva Electrophoresis) was cut into sections, activated in methanol and transferred into water. Five micrometer thick paraffin sections were cut using the microtome and collected onto the membrane. The sections were then dried for 30 min at 55 °C. Deparaffinization was done in 100 % Xylol for 10 min followed by rehydration through an ethanol series (100 %, 95 %, 70 %) for 5 min each. Membranes were washed with TBS for 5 min and digested using 10 μg/ml Proteinase K at 55 °C in PK digest buffer (10 mM Tris HCl pH7.8, 100 mM NaCl, 0.1 % Brij) for 15 h. Membranes were washed for three times in TBS-T for 5 min and then incubated in 3 % H_2_O_2_ for 5 min to block endogenous peroxidases. After a further wash step denaturation with 4 M guanidine isothiocyanate in 10 mM Tris-HCl pH 7,8 was done for 15 min to retrieve epitopes.

Membranes were blocked in 0.2 % casein dissolved in TBS-T (i-Block) for 60 min at room temperature and then incubated with primary anti-α-synuclein antibody (1:1000 dilution, BD Cat.no. 610787) overnight at 4 °C in a wet chamber. After 3 washes with TBS-T, membranes were incubated with biotinylated secondary antibody (1:500 in i-Block) for 1 h at room temperature. Detection was done after several washes using the Avidin/Biotin Technology (Vectastain Elite ABC Kit, Vector Laboratories). Reaction was stopped by washing the membranes in distilled water.

### Determination of telomere length by quantitative fluorescence *in situ* hybridization

Telomere lenght was analyzed in the brainstem, where most of the studies were performed, using a previously described protocol [48]. In short, tissues were digested in prewarmed pepsin solution (100 mg of pepsin, 100 ml of H_2_O, 84 ml of conc. HCl) for 15 min at 37 °C and washed for three times with PBS. After dehydration series, slides were dried and hybridization mix was added (1 mM Tris pH 7.2, 25 mM magnesiumchloride, 9 mM citricacid, 82 mM disodiumhydrogenphosphate, 70 % μl formamide deionized, 25 μg/ml peptide nucleic acid (PNA) probe (Panagene), 5 % blocking reagent). Slides were covered and denatured for 3 min at 80 °C, followed by an incubation step for 2 h in a humidified chamber at room temperature. After two 15 min washes with wash buffer (70 % formamide, 10 mM Tris pH 7.2, 0.1 % BSA), slides were washed with TBS-T and PBS, each twice for 5 min. Neurons were counterstained with Cy5-Neuron N dye. Afterwards, slides were dehydrated through an ethanol series and air dried. Slides were covered using VECTASHIELD. Antifade Mounting Medium with DAPI (Vector Laboratories). Neuronal cells were counterstained with Deep-Red fluorescent Nissl stain dye (Neuro Trace 640/660, ThermoFisher).

For analysis, 50 sections of the brainstem were captured using a 100 x objective and 100 nuclei were analyzed for their telomeric length, using TFL-Telo V1.0 software [49]. Five mice per group were analyzed for telomere length.

### Immunohistochemical analysis of brain tissue

Brain from transgenic mice and appropriate controls were halved and fixed in 4 % para-formaldehyde and embedded in paraffin. Five micrometer sections were cut longitudinally and after deparaffinization and rehydration, sections were boiled in 0.01 M citrate buffer pH 6.0 for 5 min. Blocking was performed in TBS-T with 1 % BSA. Slides were incubated with antibody overnight in PBS-T + 1 % BSA (1:1000, anti-Iba-1 (Wako 019-19741) and anti-α-synuclein (phospho S129) antibody (Abcam, ab51253)) at 4 °C. After three washes in PBS for 5 min, the sections were incubated with fluorescence labeled secondary antibodies for 90 min (anti-rabbit, Cy3, 1:2000, anti-mouse, Cy3 1:2000). Analysis was performed using a fluorescence microscope. For microglia analysis ten sections from brainstem were analyzed. For phospho-α-synuclein images were captured of the brainstem, cerebellum and deep mesencephalic nucleus.

### Quantification of p-asyn staining

Quantification of p-asynuclein staining was performed using ImageJ. Image acquisition was performed with 20x of magnification. Seven pictures were taken per mouse with a distance of 0.2 μm in the region of the deep mesencephalic nucleus. Microscopy data were processed and analysed using ImageJ64 1.49 software and a Macro written for ImageJ. First step of the macro was to substract the background colour of the picture and convert it into black and white. Second step was the measurement of the stained area (black), using the tool “analyze particles”. An automated threshold has been used which detected a minimum of 12 and the highest 255 particles.

### Quantitative real-time PCR analyses

A piece of brainstem was cut from the brain hemispheres were snap frozen in liquid nitrogen and stored at −80 °C until use. A piece of brainstem was cut. Disruption and homogenization of the tissue was done using the TissueLyser (Qiagen). RNA was isolated with the RNeasy lipid tissue kit (Qiagen) according to the manufacturer’s protocol. cDNA synthesis was done from total RNA using the GoScript^TM^ Reverse Transcription System (Promega). Quantitative real-time PCR analysis was done with the Absolute qPCR ROX Mix (Thermo Scientific) and the Universal Probe Library (Roche) on an ABI7300 Real-Time PCR System (Applied Biosystems). Brainstems from Terc^+/+^ mice were used as a reference. Primers were generated intron-spanning and primer sequences are mentioned in Additional file 1: Table S2.

### Morphometric analysis of reconstructed microglia

IF stained sections were analyzed by confocal laser scanning microscopy using a ZEISS LSM 510 META. High magnification and z-stack images were obtained using a LD LCI Plan-Apochromat 25x/0.8 Imm. Korr. DIC objective (Zeiss). Imaging speed was 4 (pixel dwell 12.8 μs) with a resolution of 1024x1024 pixels. For 3D-volumes to analyze microglia morphology a z-stack of 30 μm thickness with an interval of 0.8 μm was used.

Three-dimensional (3D)-reconstructions were performed using IMARIS Filament Tracer (www.bitplane.com), as previously described [23]. The z-stack was uploaded to the IMARIS-program rendering a 3D volume. Cells were reconstructed from the inferior molecular layer. Tracing was performed in a region of interest comprising only one cell. Cells were appropriate for the analysis when the staining was distinct and the whole cell including all processes was visible in the 3D volume. The automatic detection mode was applied. Parameters were: no loops allowed, start and end points calculated via spot detection. The parameters total process length, total volume, number of branch points, number of segments, number of terminal points and were analyzed. An automated Sholl analysis was also performed on each digitized cell with the IMARIS software using spheres whose radii were increasing by 1 μm per step. Five Iba1-positive microglia per animal/section were reconstructed and analyzed, and 4–5 animals were included in each group.

### Isolation of microglia from brainstem

Microglia were acutely FACS-isolated from the brain stem as described previously using CD45 and CD11b antibodies [50]. RNA was extracted from the acutely isolated microglia and using the RNeasyMicro kit (Qiagen) according to the manufacturer’s protocol isolated using Qiagen RNeasy.

### RNA sequencing and bioinformatics

RNA quality was determined by the Experion™ Automated Electrophoresis System, and samples with a minimum RIN quality score of minimally seven were used. The sequence libraries were prepared with the Illumina Truseq RNA sample preparation, and 50 bp single read sequencing was performed on the Illumina Hiseq 2500 platform. Reads were aligned using the Star 2.3.1 l aligner [51] to the ensemble reference, in which two mismatches were allowed. The aligned reads were sorted by Samtools version 0.1.19 [52] and quantified by HT-seq count 0.5.4 [53]. Data was analyzed using BioConductor packages and R, with particular importance of EdgeR [54]. Heatmaps were generated with heatmap2 function of package gplots. Gene enrichment and annotation analyses were performed using DAVID [55] and Ingenuity pathway analysis (IPA).

### Statistical analysis

Differences between groups in the experiments were evaluated for statistical significance by using the Mann-Whitney *U* test (for parameters measured at discrete time-points, non-parametric test) or the Log-rank Mantel-Cox test (Kaplan-Meier curves). Differences with P values of less than 0.05 were considered significant. Statistical analysis of beamwalk were performed using the 2-way anova test. Analyses were conducted using the GraphPad Prism software, version 5.04.

## Results

### Telomere shortening reduces the life span of α*-*synuclein transgenic mice

**Fig. 1:**
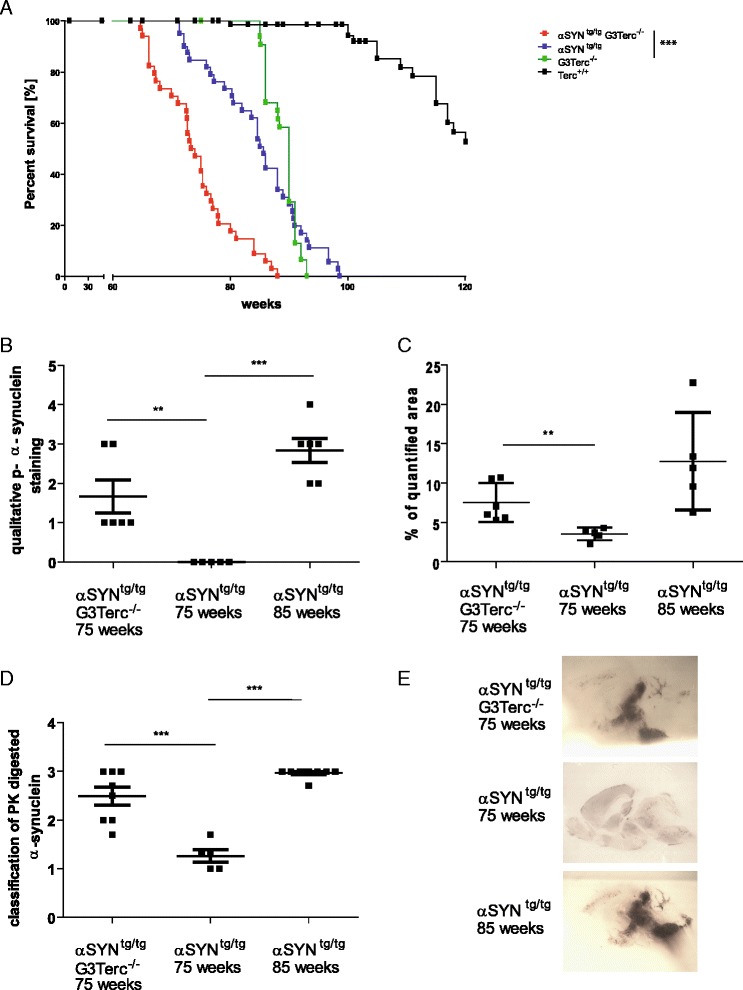
Telomere shortening shortens lifespan of αSYN transgenic mice and increases aggregate formation. **a** Kaplan Meier survival curves for αSYN^tg/tg^ G3Terc^-/-^ (*n* = 34), αSYN^tg/tg^ (*n* = 36), G3Terc^-/-^ (*n* = 31) and Terc^+/+^ mice (*n* = 21). αSYN^tg/tg^ G3Terc^-/-^ mice show a significant decrease in survival (median life span: 73.6 weeks) in comparison to the αSYN^tg/tg^ mice (median life span 85.6 weeks; *p* < 0.0001) and the G3Terc^-/-^ mice (median life span 90 weeks; *p* < 0.0001). **b** Analysis of the pathological α-synuclein phosphorylation (p-αsyn). The amount of the p-αsyn used as a marker of disease severity. To characterize the severity and the affected zone, a scoring system was used (see Additional file 3: Figure S2). Stainings were repeated and evaluated three times and the mean values of the scores were used. αSYN^tg/tg^ G3Terc^-/-^ mice (75 weeks old with a motoric phenotype) showed significantly more p-αsyn staining than age-matched αSYN^tg/tg^ mice (75 weeks old, no phenotype, *p* = 0.0064). **c** Quantification of p-asynuclein staining in the region of deep Mesencephalic nucleus. Seven sections of the region were taken and quantified using ImageJ. αSYN^tg/tg^ G3Terc^-/-^ mice show significant higher p-asynuclein staining in comparison to age matched αSYN^tg/tg^ mice (*P* = 0.0043). **d** Classification of Proteinase K resistant α-synuclein aggregates. αSYN^tg/tg^ G3Terc^-/-^ mice showed stronger aggregate formation compared to αSYN^tg/tg^ mice (75 weeks old, no phenotype, *p* = 0.0006). 85 weeks old αSYN^tg/tg^ mice with terminal phenotype showed a very dense pattern of p-αsyn positive aggregates (αSYN^tg/tg^ 75 weeks vs. αSYN^tg/tg^85 weeks; *p* < 0.0001). **e** Representative pictures of membranes, where whole brain hemispheres were attached and digested with Proteinase K. Proteinase K resistant α-synuclein aggregates reflect the severity of disease

In order to investigate the effects of ageing in the Parkinson’s disease mouse model, Thy-1 h[A30P] α–synuclein transgenic mice (αSYN^tg/tg^) were crossed with Terc knockout mice (Terc^-/-^). For the final study cohorts, the 3rd generation Terc^-/-^ mice with short telomeres were generated (G3Terc^-/-^), with or without the human mutated [A30P] α–synuclein transgene (αSYN^tg/tg^ G3Terc^-/-^ and G3Terc^-/-^ Additional file 2: Figure S1A). Mice with wild type Terc were used as controls (αSYN^tg/tg^ and Terc^+/+^; Additional file 2: Figure S1A). Cohorts of 75 weeks old G3Terc^-/-^ animals showed a significant, age-dependent reduction in telomere length in the brainstem (Additional file 2: Figure S1B). αSYN^tg/tg^ mice are known to develop an obvious motoric phenotype at 80–85 weeks of age, which first affects hind limb mobility, showing a weakening of extremities and influence on the locomotor performance [47]. This motoric phenotype occurs due to the loss of neurons and Lewi body-like inclusions in the different compartments of the brain [42]. Telomere dysfunction led to a dramatic reduction of life span. αSYN^tg/tg^ G3Terc^-/-^ animals died significantly earlier with a median life span of 73.6 weeks, whereas αSYN^tg/tg^ animals survived with a median of 85.6 weeks (Fig. 1a, *p* < 0.0001, Log-rank (Mantel-Cox) Test).

### Telomere shortening is associated with progression of the disease-related aggregate formation in Thy-1 [A30P] α*-*synuclein transgenic mice

α–Synuclein is located in the presynaptic neurons and accumulated with progressive disease. After undergoing posttranslational modification, phosphorylation of α–synuclein at serine129 serves as a disease progression marker [56, 57]. In order to investigate whether the earlier onset of synucleinopathy in αSYN^tg/tg^ G3Terc^-/-^ animals was due to accelerated aggregate accumulation, phosphorylated α-synuclein on Serin129 was analyzed by phospho-α–synuclein staining and aggregate formation measured using PK-PET Blot. Accordingly, the 75 weeks old αSYN^tg/tg^ G3Terc^-/-^ animals showing a motoric phenotype were compared with 75 weeks old αSYN^tg/tg^ animals without phenotype as well as with phenotypic αSYN^tg/tg^ mice with a median age of 85 weeks. Comparison was done using a score as shown in Additional file 3: Figure S2.

Analysis of the brainstem revealed a significantly higher amount of phosphorylated α-synuclein in αSYN^tg/tg^ G3Terc^-/-^ mice compared to the aged-matched group of αSYN^tg/tg^ mice (Fig. 1b-e and Additional file 3: Figure S2A, *P* = 0.0064). Eighty-fiveweeks old αSYN^tg/tg^ mice showed an increase in phosphorylated α-synuclein (Fig. 1b, *P* < 0.0001, Additional file 3: Figure S2A). Quantification of p-asyn staining in deep mesencephalic nucleus using ImageJ showed significant differences between αSYN^tg/tg^ G3Terc^-/-^ animals 75 weeks old αSYN^tg/tg^ (Fig. 1c, *P* = 0.0043).

Thus, telomerase dysfunctional αSYN^tg/tg^ G3Terc^-/-^ mice at 75 weeks showed an increased aggregate formation in comparison to the age-matched αSYN^tg/tg^ mice, and 85 weeks old αSYN^tg/tg^ mice displayed the highest aggregate formation. Similar observations were made with the classification of PK digested α-synuclein, whereas αSYN^tg/tg^ G3Terc^-/-^ mice compared to the aged-matched group of αSYN^tg/tg^ mice showed significant higher levels of proteinase K resistant aggregates (Fig. 1d, αSYN^tg/tg^ G3Terc^-/-^ vs. αSYN^tg/tg^ Terc^+/+^; *P* = 0.0006 & Fig. 1e). Definition of the score, which was used to assess proteinase K digested aggregates is shown in Additional file 3: Figure S2B.

### Telomere shortening deteriorates motor balance and coordination in Thy-1 [A30P] α*-*synuclein transgenic mice

**Fig. 2:**
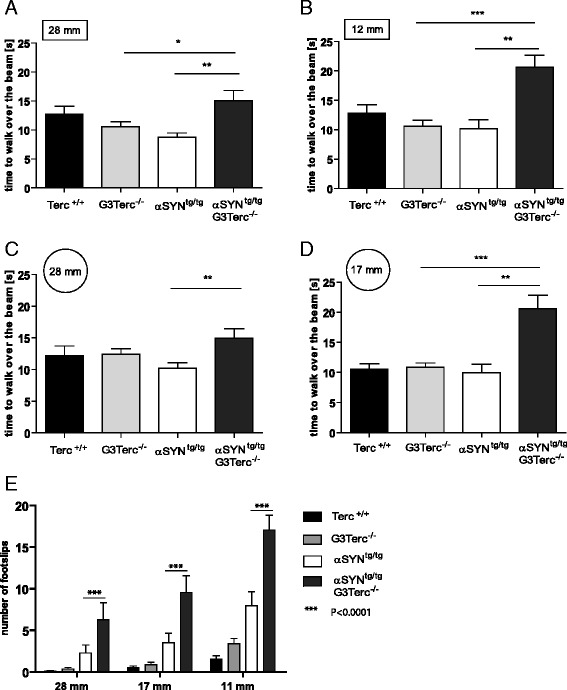
Behavioural characterization of αSYN^tg/tg^ transgenic mice in the context of telomere shortening. Behavioral experiments were performed before the occurrence of a motoric phenotype. All mice were tested at an age of 72 weeks (*n* = 7–10). **a**-**d** Beamwalk: Mice were trained for three consecutive days to walk over a wooden beam. The test was performed with different beam diameters and shapes (square or round). Graphs show the latency to walk over a beam with a square diameter of **a** 28 mm (αSYN^tg/tg^ G3Terc^-/-^ mice vs. αSYN^tg/tg^; *p* = 0.0095), **b** 12 mm (αSYN^tg/tg^G3Terc^-/-^ mice vs. αSYN^tg/tg^; *p* = 0.0023) or a round beam with a diameter of **c** 28 mm (αSYN^tg/tg^ G3Terc^-/-^ mice vs. αSYN^tg/tg^; *p* = 0.0095), and **d** 17 mm (αSYN^tg/tg^ G3Terc^-/-^ mice vs. αSYN^tg/tg^; *p* = 0.003). αSYN^tg/tg^ G3Terc^-/-^ mice showed a reduced latency to cross the different beams. **e** Number of footslips. αSYN^tg/tg^ G3Terc^-/-^ mice show a significant increase in the number of footslips in comparison to αSYN^tg/tg^ mice (*p* < 0.0001; 2-way Anova)

As previously described, αSYN^tg/tg^ transgenic mice develop a progressive loss of motor balance, correlating with the accumulation of aggregates [43]. To determine the influence of telomere dysfunction on motor balance, general balance and coordination, 72 weeks old mice from the different cohorts were compared using the beamwalk test. This test is used to assess the motor phenotype in mouse models [45, 58]. We analyzed the number of paw slips and the time which was needed to traverse over beams of different sizes and shapes (Fig. 2a -e).

αSYN^tg/tg^ G3Terc^-/-^ mice required more time to cross the beam than αSYN^tg/tg^ mice (Fig. 2a), which was observed for all four beam types tested (Fig. 2a-d). The absolute number of foot slips while crossing the beam, was counted over all walks. αSYN^tg/tg^ G3Terc^-/-^ mice showed a significant increase in the number of footslips compared to αSYN^tg/tg^ mice (Fig. 2e; *P* < 0.0001, 2-way Anova). Furthermore, exploratory behavior, especially the activity and the duration in different zones of the open field showed no significant difference between the different groups (Additional file 4: Figure S3A & B), in line with previous data showing that the α-synuclein phenotype declines cognitive effects and coordination [47, 59], but not exploratory and anxiety-related behavior of the animals. Also, the forepaw grip capacity using the string agility test did not reveal any significant differences at the time of the experiment (Additional file 4: Figure S3C).

### Telomere shortening is associated with a decline in activation of microglia in brainstem

**Fig. 3:**
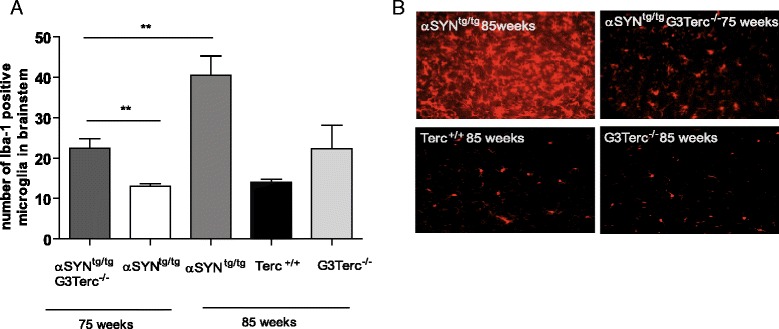
Activation of microglia and astrocytes in the brainstem. **a** The histogram represents the number of Iba-1 positive microglia in the brainstem (*n* = 5–6 mice per group, ten pictures were counted per mouse). Note that aged-matched αSYN^tg/tg^ G3Terc^-/-^ mice (average 22.47 ± 2.32, *n* = 6) and αSYN^tg/tg^ mice (average 13.00 ± 0.67, *n* = 5) show a significant difference in number of microglia (*p* = 0.0058). **b** Representative pictures of Iba-1 staining in brainstem

Microgliosis is a well-known process in brain pathology and has been described in all animal models of PD [60, 61] and in PD patients [62]. We therefore examined the expression of the microglia marker Iba-1 in our various mouse cohorts. While no significant differences in Iba-1 immunoreactivity were found in mice without phenotypic synucleinopathy, the 75 week old αSYN^tg/tg^, 85 week old Terc^+/+^ animals and 85 week old G3Terc^-/-^ all displayed similar number of Iba1 positive cells (Fig. 3a). The presence of behavioral phenotypes were accompanied by an induction of Iba-1 immunoreactivity in both 75 week old αSYN^tg/tg^ G3Terc^-/-^ and 85 week old αSYN^tg/tg^animals (Fig. 3a; *P* = 0.0058 and Fig. 3b). Interestingly, Iba-1 immunoreactivity was significantly higher in 85 weeks old αSYN^tg/tg^ with phenotype compared to 75 week old αSYN^tg/tg^ G3Terc^-/-^ mice with phenotype (Fig. 3a; *P* = 0.0072). These results support the hypothesis that telomere erosion/shortening may lead to an impaired microglia response.

**Fig. 4:**
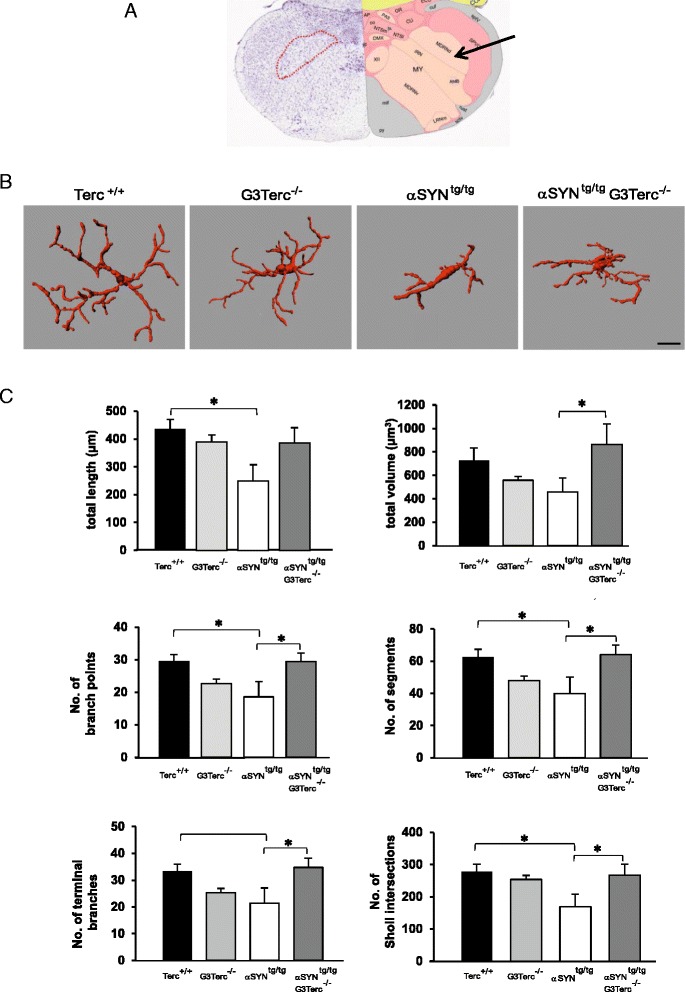
3D reconstruction of brainstem microglia and their morphometric analysis. **a** Coronal section through the medulla outlining the region from which Iba1-positive microglia were selected for 3D morphometric analysis (*arrow*, right; *broken red line*, left) MDRN: Medullary reticular nucleus, dorsal part. Image reproduced from the Allen Brain Atlas. **b** Representative 3D-reconstructed microglia from aged WT (Terc^+/+^), G3Terc^-/-^, αSYN^tg/tg^ mice and αSYN^tg/tg^ G3Terc^-/-^ mice. **c** Morphometric analyses of reconstructed microglia. Six different parameters relating to process length and branching were analysed using Imaris Bitplane software. For a more detailed description of the measurement characteristics, see Additional file 5: Figure S4. Five Iba1-positive microglia per animal were reconstructed and analyzed, and 4–5 animals were included in each group. Mean group values + SEM are depicted. A one-way ANOVA with a post-hoc LSD test was applied to determine significant differences between groups. *p < 0.05. **b** Scale Bar = 10 μm

To further address and confirm the differences in microglia response a detailed 3D morphological analysis was performed. For this purpose only microglia in the dorsal part of the medullary reticular nucleus (MDRNd) of the brain stem were analyzed to exclude potential morphological differences due to the brain region (Fig. 4a). Representative pictures of single cells from 75 week old αSYN^tg/tg^ G3Terc^-/-^ mice and 85 week old wild type animals (Terc^+/+^), G3Terc^-/-^ and αSYN^tg/tg^ revealed a significant morphological microglia response in 85 week old αSYN^tg/tg^ only (Fig. 4b). In all other mice, microglia displayed a more ramified morphology reminiscent of a non-reactive microglia phenotype (Fig. 4b). This was confirmed by quantitative morphometric analysis for total process length, volume of processes, number of process branch points, number of process segments, number of terminal process branches and number of Sholl intersections (Fig. 4c, Additional file 5: Figure S4 for methodological details). All these readouts confirm that only microglia in symptomatic, 85 week old αSYN^tg/tg^ animals significantly changed their morphology. Interestingly, this morphological transition was not observed in affected 75 week old αSYN^tg/tg^ G3Terc^-/-^ mice, furthermore suggesting that telomere attrition impaired the microglia response in the presence of PD pathology (Fig. 4c).

### Telomere shortening is linked to a reduced expression of inflammatory genes

**Fig. 5:**
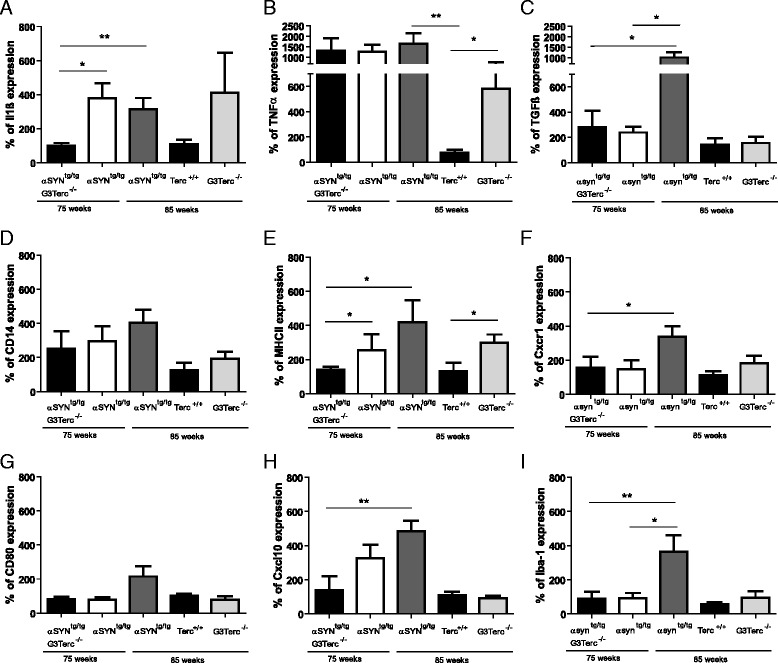
Expression level of inflammatory markers. RT-PCR was performed on the inflammatory markers **a** Il1ß (αSYN^tg/tg^ G3Terc^-/-^ mice vs. αSYN^tg/tg^; *p* = 0.0147), **b** TNFα and **c** TGFß. Inflammation-induced activation of monocytes was shown with CD14 (**d**), MHC2 (**e**) and CXCR1 (**f**). Monocyte activation was analyzed with CD80 (**g**) and Interferon γ induced protein CXCL10 (**h**). **i** Iba1 was clearly reduced in αSYN^tg/tg^ G3Terc^-/-^ mice (αSYN^tg/tg^ G3Terc^-/-^ mice vs. αSYN^tg/tg^; *p* = 0.0202). After substracting of the housekeeping gene all different groups were normalized to the average ct of the Terc^+/+^ group

The lack of a morphological response of microglia in diseased mice with short telomeres prompted the question whether the inflammatory response in α-synuclein transgenic mice with and without telomerase function was different. We therefore compared the mRNA expression of different inflammatory markers and chemokines in whole brainstem RNA of the different subgroups (Fig. 5). For normalization, the house keeping gene was subtracted and the Terc^+/+^ group were set to 100 %. The pro-inflammatory cytokine IL-1ß was upregulated in 75 and 85 weeks old αSYN^tg/tg^ mice irrespective of the presence of disease symptoms. However, this cytokine was significantly lower expressed in αSYN^tg/tg^ G3Terc^-/-^ compared to αSYN^tg/tg^ mice (Fig. 5a; *p* = 0.0147)). Other central inflammatory markers like TNFα or TGFß were highly elevated in 85 weeks old αSYN^tg/tg^ mice with a clear phenotype, but this increase was not detected in αSYN^tg/tg^ G3Terc^-/-^ mice with phenotype (Fig. 5a-c). Similar expression patterns were observed for *CD14, MHCII, CXCR1*, and *CXCL10* (Fig. 5d -h). Iba-1 RNA expression levels confirmed that symptomatic αSYN^tg/tg^ 85 week old animals had the highest expression level of Iba-1 (Fig. 5i: αSYN^tg/tg^ G3Terc^-/-^ compared to αSYN^tg/tg^ 85 weeks; *P* = 0.0202). The data suggested an inflammatory reaction in symptomatic αSYN^tg/tg^ 85-week-old animals which was impaired in Terc-deficient animals.

Similarly, reactive astrocytes (determined by staining for glial fibrillary acidic protein (GFAP) were significantly increased in phenotypic αSYN^tg/tg^ animals at 85 weeks of age and with distinct phenotype (Additional file 4: Figure S3D). Mice with short telomeres at their final stage of disease show reduced astrocyte activation in comparison to αSYN^tg/tg^ with disease (Additional file 4: Figure S3D).

### RNA-sequencing analysis of brain stem microglia indicate partially opposing gene expression programs

To determine how telomere erosion affected microglia gene expression in αSyn^tg/tg^ mice, brainstem microglia were FACS sorted from αSyn^tg/tg^ and αSyn^tg/tg^ G3Terc^-/-^ mice, and gene expression levels were quantified using RNA sequencing.

**Fig. 6:**
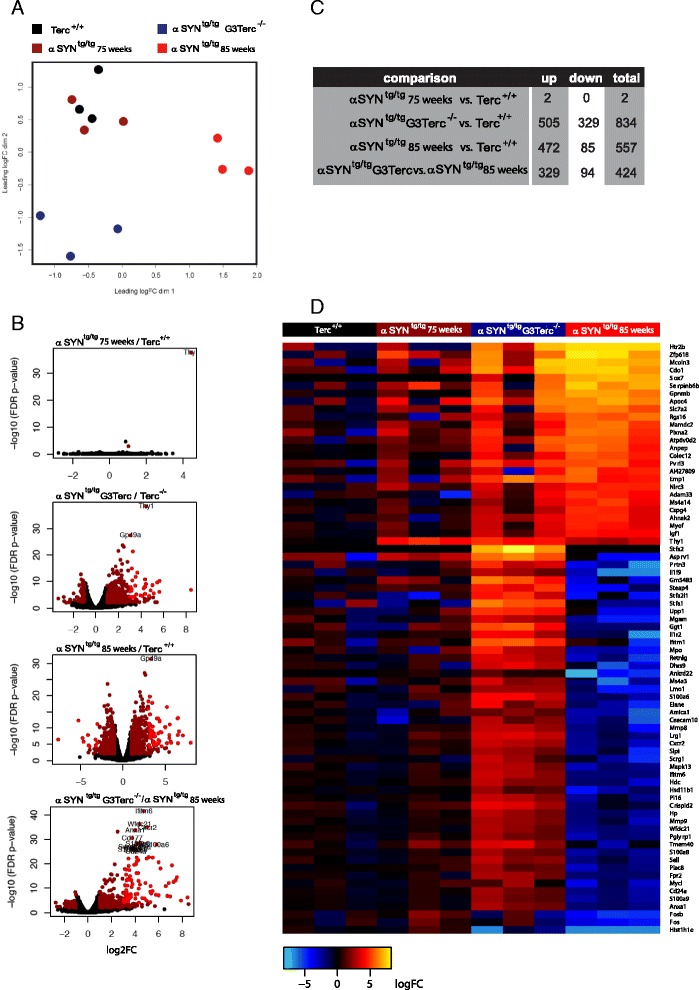
RNA sequencing analysis of microglia. **a** Multidimensional scaling plot of microglia RNAseq data from different mice. αSYN^tg/tg^ microglia from mice with a phenotype cluster separately from all other microglia samples and are most similar to αSYN^tg/tg^ G3Terc^-/-^ microglia. Gene expression in control and αSYN^tg/tg^ microglia without a phenotype are very similar and form a separate, mixed cluster. **b** Volcano plots showing the significantly differentially expressed genes between pairs of conditions. *Dark red dots*: FDR <0.05 and logFC >1 and *bright red dots*: FDR <0.0001 and logFC >3. **c** The number of differentially expressed genes (up, down and total) between the compared groups. **d** Heatmap visualization of genes differentially expressed between αSYN^tg/tg^ and αSYN^tg/tg^ G3Terc samples (FDR <0.001, logFC >3)

Multidimensional scaling analysis showed clustering of samples in a genotype- and phenotype dependent manner (Fig. 6a). Microglia samples from control mice and unaffected αSYN^tg/tg^ mice clustered closely together. Microglia samples from diseased αSYN^tg/tg^ mice clustered most distant from all other samples. Interestingly, the samples isolated from affected αSYN^tg/tg^ G3Terc^-/-^ mice clustered separately but closer to control microglia than αSYN^tg/tg^ samples.

Gene expression profiles were analyzed and pair-wise comparisons were plotted, with dark red: FDR <0.05 and logFC >1 and bright red: FDR <0.0001 and logFC >3 as criteria (Fig. 6b). The gene expression profiles of control and unaffected αSYN^tg/tg^ microglia were almost indistinguishable; only 2 genes were differentially expressed between control and unaffected αSYN^tg/tg^ microglia (Fig. 6b, common criteria FDR <0.05 and logFC >1). Gene expression in microglia isolated from αSYN^tg/tg^ mice that displayed disease phenotype (αSYN^tg/tg^ phenotype) was very different from control microglia with 834 genes were differentially expressed (Fig. 6c). An intermediate gene expression profile was observed in αSYN^tg/tg^G3Terc^-/-^ mice with 557 genes were differentially expressed between αSYN^tg/tg^ G3Terc^-/-^ and control microglia (Fig. 6c). These data indicate that microglia in diseased αSYN^tg/tg^ G3Terc^-/-^ behave differently in terms of gene expression changes in gene expression compared to affected αSYN^tg/tg^ microglia with regard to αsyn-induced pathology. Comparison of αSYN^tg/tg^ and αSYN^tg/tg^ G3Terc^-/-^ microglia expression profiles showed that 423 genes were differentially expressed between these samples and that most of these genes (329) had an increased expression in αSYN^tg/tg^ G3Terc^-/-^ microglia (Fig. 6c), suggesting that the lack of telomerase function had a significant influence on the microglia response to α-synuclein pathology. The number of differentially expressed genes between the compared samples and if they are up- or down-regulated is indicated in Fig. 6c.

To visualize differences in gene expression between control, unaffected and affected αSYN^tg/tg^, and αSYN^tg/tg^ G3Terc^-/-^mice, a heat map of the most significantly (FDR <1E-3, logFC >3) differentially expressed genes between these samples was generated (Fig. 6d). Many of the genes upregulated in both SYN^tg/tg^ G3Terc^-/-^ and αSYN^tg/tg^ microglia were more abundantly expressed in αSYN^tg/tg^ microglia. Besides genes that were similarly up- or down-regulated in both data sets, several genes were antagonistically expressed between αSYN^tg/tg^ G3Terc^-/-^ and αSYN^tg/tg^ microglia with many genes that were down-regulated in expression in αSYN^tg/tg^ microglia showed an increased expression in αSYN^tg/tg^ G3Terc^-/-^ microglia. MMP8, MMP, CXCR2, IL1R1, S100A and S100B were downregulated in αSYN^tg/tg^ microglia and upregulated in αSYN^tg/tg^ G3Terc^-/-^ microglia.

Next, Ingenuity Pathway Analysis (IPA) was used for enrichment analyses of genes with increased in expression in both αSYN^tg/tg^ G3Terc^-/-^ and αSYN^tg/tg^ samples and of genes with increased expression in αSYN^tg/tg^ G3Terc^-/-^ compared to αSYN^tg/tg^ microglia to identify pathways that were overrepresented in these gene lists (Table 1). There were several pathways similarly regulated in both αSYN^tg/tg^ G3Terc^-/-^ and αSYN^tg/tg^ samples, for example Granulocyte Adhesion and Diapedesis or Atherosclerosis Signaling (Table 1). However, prominent differences concerning predicted pathways activity were also observed between αSYN^tg/tg^ G3Terc^-/-^ and αSYN^tg/tg^ samples. As shown in Table 1 LXR/RXR activation was significantly enriched in the downregulated profile in αSYN^tg/tg^ G3Terc^-/-^ but significantly up-regulated in αSYN^tg/tg^ samples. Both cAMP-mediated signaling and acute phase response signaling were significantly inhibited in αSYN^tg/tg^ samples only, whereas cell cycle events were only significantly inhibited only in αSYN^tg/tg^ G3Terc^-/-^ microglia. These cells significantly upregulated the pathway for nitric oxide and reactive oxygen species production, whereas no such response was found in αSYN^tg/tg^ samples. Significantly changed IL-10 signaling was only found in αSYN^tg/tg^ G3Terc^-/-^ microglia, whereas αSYN^tg/tg^ samples showed significant changes in G-protein coupled receptor signaling. However, the data of the latter two did not allow drawing conclusions about the direction of these changes.

**Table 1:** Changes in IPA canonical pathways in brainstem microglia

IPA canonical pathway	αSYN^tg/tg^ G3Terc^-/-^vs. controls (*p* value)	Predicted activity pattern (z score)	αSYN^tg/tg^ vs controls (*p* value)	Predicted activity pattern (z score)
Granulocyte Adhesion and Diapedesis	5,75E-08	NA	2,34E-09	NA
Atherosclerosis Signaling	7,76E-07	NA	4,27E-07	NA
LXR/RXR Activation	8,51E-08	Inhibition-0,277	9,55E-09	Activation 2,5
cAMP-mediated signaling	ns	NA	7,59E-06	Inhibition-0,688
Acute Phase Response Signaling	ns	NA	0,000104713	Inhibition-0,535
Cell Cycle: G2/M DNA Damage Checkpoint Regulation	3,89E-05	Inhibition-0,378	ns	NA
Production of Nitric Oxide and Reactive Oxygen Species in Macrophages	1,02E-05	Activation 2,496	ns	NA
IL-10 Signaling	4,57E-05	NA	ns	NA
G-Protein Coupled Receptor Signaling	ns	NA	8,51E-06	NA

## Discussion

We here show that telomere shortening correlated to an accelerated phenotype and early death in our Parkinson mouse model. Moreover, our data suggest that telomere shortening inhibited a potentially protective inflammatory microglia response with respect to αSyn pathology. The present study adds up to an ongoing discussion that telomeres and telomere erosion may play a pivotal role in Parkinson’s disease.

It is very well known that microglia are activated in all synucleopathies including humans with PD [62–64]. Numerous reports have described the expression of pro-inflammatory cytokines in microglia in PD pathology. It has been described that αSyn itself is a powerful inducer of microglial IL-1β expression and release, which includes TLR2 signaling and NLRP3 inflammasome activity [65, 66]. Usually, this pro-inflammatory function of microglia is suggested to actively contribute to PD pathology [67, 68] and microglia have often been discussed being detrimental elements in PD [69, 70].

The data presented here clearly confirm the pro-inflammatory response of wild type microglia in the presence of αSyn pathology. Microglia in the αSYN^tg/tg^ model showed the classical morphological signs of activation such as retraction of processes and we furthermore detected changes in mRNA of several pro-inflammatory markers in the brain stem of these mice. Surprisingly, immunohistochemical analysis of Iba1 expression and a detailed analysis of microglia morphology suggested that microglia with short telomeres displayed an impaired response in the presence of αSyn pathology in our αSYN^tg/tg^ model of PD. This impaired microglia response was furthermore supported by qPCR investigations as none of the investigated pro-inflammatory markers was increased in the brainstem of αSYN^tg/tg^ G3Terc^-/-^ animals. In addition to the impaired inflammatory microglia response, αSYN^tg/tg^ G3Terc^-/-^ animals showed accelerated disease progression and a significantly reduced life span. Our data suggest that the microglia response in the αSYN^tg/tg^ model of PD may not be solely detrimental, but may also have protective elements inhibiting disease progression and early death.

Indeed it was described that microglia showed an enhanced phagocytic activity when treated with monomeric αSyn [68]. Microglial cells were capable of fast αSyn degradation with an intracellular αSyn half-life of about 4 h, which is about half of the degradation time for αSyn in astrocytes and neurons [71]. Regarding microglial phagocytosis of αSyn, Toll-like receptors (TLRs) seem to play an important part in recognition and internalisation of αSyn via TLR activity [71]. Accordingly, it was demonstrated recently that TLR4 ablation leads to a disturbed clearance of αSyn by microglia [72]. Thus, microglia were important factors in the clearance of toxic αSyn in the brain. Our observation that in the brains of αSYN^tg/tg^ G3Terc^-/-^ animals αSyn pathology was significantly increased when microglia response was impaired would corroborate this notion.

Why microglia in αSYN^tg/tg^ G3Terc^-/-^ animals failed to morphologically react to abundantly present αSyn in the brain is unclear at the moment. Our gene expression analysis did not show differences between G3Terc^-/-^ and wild type microglia suggesting that lack of telomerase activity and telomere shortening did not have a major impact on microglia gene expression pattern in general. Similarly, when comparing the response of microglia with and without telomere attrition to peripheral LPS injections, no differences were observed, suggesting that microglia in G3Terc^-/-^ animals are not generally impaired in their inflammatory reactivity [73].

To understand the molecular responses of microglia with long and short telomeres to the presence of αSyn pathology we subjected brain stem microglia to RNAseq and bioinformatical analysis. These data revealed that the reaction of microglia in αSYN^tg/tg^ G3Terc^-/-^ animals was not blunted. Thus despite no obvious changes in morphology, αSYN^tg/tg^ G3Terc^-/-^ microglia did show changes in gene expression. Microglia in αSYN^tg/tg^animals with phenotype up- and down-regulated 505 and 329 genes compared to controls, respectively. Microglia in αSYN^tg/tg^ G3Terc^-/-^ animals with phenotype showed less changes, as these cells up-regulated 472 genes but only 85 genes were found downregulated. Of the up-regulated genes, 224 were induced in both sample sets, however it was obvious that this induction was more pronounced in microglia in αSYN^tg/tg^ animals suggesting that with respect to these genes, microglia from αSYN^tg/tg^ G3Terc^-/-^ animals had a similar but less prominent response to their counterparts with long telomeres. However, 153 genes that were found up-regulated in microglia from αSYN^tg/tg^ G3Terc^-/-^ animals, were either not induced or even down-regulated in microglia from αSYN^tg/tg^ animals, thus for these 153 genes opposing expression patterns were observed suggesting that these genes may point towards differential microglia functions. It is interesting to note here in αSYN^tg/tg^ G3Terc^-/-^ microglia MMP8, MMP, CXCR2, IL1R1, S100A and S100B were upregulated suggesting that an inflammatory activation of these cells was not completely absent, as indicated by our qPCR analysis from brain stem material.

To gain more knowledge about how the microglia response was altered between αSYN^tg/tg^ G3Terc^-/-^ and αSYN^tg/tg^ microglia, IPA canonical pathway analysis was performed. It was obvious that LXR/RXR signaling was inhibited in microglia from αSYN^tg/tg^ G3Terc^-/-^ animals whereas an up-regulation of this pathway was observed in microglia from αSYN^tg/tg^ animals. LXR/RXR belong to the family of heterodimeric Type II nuclear receptors [74] which in cells of the myeloid lineage drive the acquisition of a cellular state that promotes tissue repair and phagocytosis [75]. There are various lines of evidence that LXR/RXR signaling promotes the microglial up-take of amyloid beta, decreases plaque load in mouse models of Alzheimer’s disease and improves the memory deficits of these mice [76–78]. As αSyn accumulations are found intraneuronal (see below for discussion) and microglia thus are not in direct contact with these accumulations phagocytosis of αSyn accumulations most likely is not part of the protective program of microglia with long telomeres. Our data linking increased LXR/RXR signaling and prolonged survival of αSYN^tg/tg^animals would suggest a protective function of this signaling pathway also in our Parkinson’s disease model. In such scenario wild type microglia would respond to the presence of αSyn accumulating neurons with increased LXR/RXR signaling. Whether this hypothesis holds true and how increased LXR/RXR signaling in microglia finally protects from αSyn pathology remains to be investigated.

Although much remains to be learned about the different microglia responses and their impact on αSyn pathology and the survival of animals, our data point towards an aging related dysfunction of microglia, which negatively impact neurodegeneration. Microglia in the aged brain have been suggested to be primed for activation, meaning that they acquire a state of exaggerated inflammatory reactivity and/or persistent neuroinflammation. As such microglia priming is considered an important confounding factor in age-associated neurodegenerative diseases [79, 80]. On the other hand, dystrophic microglia, characterized by loss of structural integrity, presence of spheroid inclusions and fragmented cellular processes have been reported in the aged human brain [81] or in rodent mouse models of accelerated aging and neurodegeneration [82, 83]. Since dystrophy in microglia is restricted to aged and neurodegenerative brain tissues, it has been proposed to be the consequence of age-associated telomere shortening and replicative senescence in microglia [36]. In contrast to primed microglia, dystrophic microglia have been suggested to be functionally impaired. As a direct consequence the brain becomes more vulnerable potentially leading to neurodegenerative disease [84].

Whether or not telomere shortening in vivo has a direct impact on microglia functionality is currently unclear. We previously demonstrated that telomere shortening did not influence basal microglia gene expression pattern or unchallenged microglia functions [73], which is in agreement with the here presented data. However, despite the lack of a morphological response we here also show that microglia with short telomeres displayed a clearly different reaction at the mRNA level in the presence of αSyn pathology. It was surprising to see that microglia with an indistinguishable mRNA expression pattern showed a different response towards pathology and to our knowledge such an observation has not yet been published elsewhere. The reason for this peculiar finding is not clear at the moment, but nevertheless clearly indicating that short telomeres influences microglia gene expression and functionality in response to brain pathology.

Even though telomere shorting has been described for microglia [34–36] we can not exclude effects in other cells. Our data also show that the response of astrocyte is impaired in TERC-/- mice with αSyn pathology. As there is little if any data about telomere shortening in astrocytes [34] it is unclear whether this impaired astrocyte response to directly due to the knockout of telomerase in astrocytes. More likely might be that astrocytes responded differently to the changed inflammatory reaction in TERC-/- animals. Moreover, we have recently shown differences in blood brain barrier (BBB) function in third generation TERC-/- animals resulting in increased infiltration of the brain in peripheral inflammation [73]. Although in this study BBB function or brain infiltration was not explicitly investigated, we have no data that would be in favor of such disturbance. Please note that we would have seen infiltrated myeloid cells in our microglia isolation experiments that were performed for the mRNA seq analysis.

Another peculiar finding of this study is that a-syn pathology in 85 week old αSYN^tg/tg^ animals was significantly higher than in 75 week old αSYN^tg/tg^ G3Terc^-/-^ animals. Yet, both lines showed severe motor deficits and premature death raising the question of potential a-syn independent pathological effects in 75 week old αSYN^tg/tg^ G3Terc^-/-^ animals. Generally it is not entirely clear how [A30P]αSYN pathology leads to motor deficits and death, as neuronal loss is not found in this animal model [42]. In preliminary experiments we have compared αSyn accumulations in in 85 week old αSYN^tg/tg^ animals and 75 week old αSYN^tg/tg^ G3Terc^-/-^ animals and did not find striking differences. In both mice the typical αSyn accumulations located most likely in synapses, neuronal cell bodies and neurites, similar to the published observations [42] (data not shown). However, a detailed and quantitative comparison of αSyn accumulations in in 85 week old αSYN^tg/tg^ animals and 75 week old αSYN^tg/tg^G3Terc^-/-^ animals is still pending and would be an important feature of future studies. Moreover, it remains to be established whether potential differences in tau pathology [85] may be found in the severe disease in 75 week old αSYN^tg/tg^ G3Terc^-/-^ animals despite relatively low αSyn pathology.

## Conclusions

The data presented here corroborate the assumption that human dystrophic microglia in the aged brain with shorted telomeres are dysfunctional [81, 84]. Recent studies indeed have linked telomere dysfunction to various neurological diseases, which indicates the importance of telomere erosion with ageing as a major risk factor in neurodegenerative disorders. As such, telomere length in leukocytes serves as a common predictor for neurological disease and telomere shortening has been observed in patients with dementia and Parkinson’s disease [37, 86, 87]. However, as studies on this subject are controversial [88, 89], further research is needed to elucidate the effect of telomere shortening on microglia function and its impact for the development of neurodegenerative diseases.

## Additional files

Additional file 1: Table S2. Primer sequences which were used for RT-PCR. (DOC 40 kb) Additional file 2: Figure S1. Mating scheme for the generation of mouse cohorts. (A) Breeding scheme for generating 3rd generation telomerase knockout and α-synuclein transgenic mice (αSYN^tg/tg^ G3Terc^-/-^) and corresponding control cohorts. Heterozygous α-synuclein (αSYN) mice were crossed with heterozygous telomerase knockout mice (Terc) to generate double transgenic αSYN^tg/tg^ G1Terc^-/-^ and single transgenic αSYN^tg/tg^, G1Terc^-/-^ and Terc^+/+^ mice (G1 = first generation of telomerase knockout). These double transgenic αSYN^tg/tg^ G1Terc^-/-^ mice were crossed with each other to produce αSYN^tg/tg^ G2Terc^-/-^ mice and finally αSYN^tg/tg^ G3Terc^-/-^ mice. (B) Telomere length was measured in neurons of the brainstem using qFISH telomere staining double-stained with Cy5-Neuron N dye. The graph represents telomere length in brainstem in 75 weeks old mice (*n* = 5 mice per group). Analyzed were αSYN^tg/tg^ G3Terc^-/-^ in comparison to αSYN^tg/tg^ mice (*P* = 0.0012) and G3Terc^-/-^ in comparison to Terc^+/+^ mice (*P* = 0.0079) as well as αSYN^tg/tg^ G3Terc^-/-^ compared with G3Terc^-/-^ (*P* = 0.03). (PDF 29 kb) Additional file 3: Figure S2. Classification and scoring of phospho-α-synuclein and PK-PET Blot. (A) Classification of phospho-α-synuclein staining into four different scores. Representative pictures for scoring. Score 0: no p-α-synuclein staining, score 1: little staining in brainstem and DpMe, score 2: strong staining in brainstem and DpMe, score 3: strong p-α-synuclein staining in brainstem, DpMe, and cerebellum indicating severe disease progression. (B) Scoring to classify PK-PET Blot. Score 0: no PK resistant aggregates, score 1: light aggregates in brainstem and Deep Mesencepahlic nucleus (DpMe), score 2: clear PK resistant aggregates in brainstem and DpMe, score 3: dominant aggregates in brainstem and DpMe. Score 4: Prominent aggregates in brainstem, DpMe and cerebellum. (PDF 125 kb) Additional file 4: Figure S3. Exploratory behavior and string agility. Exploratory behavior of the mice has been analysed using the Open Field System. Several parameters, e.g. the activity, duration and the totally travelled distance were measured in three different zones of the open field: Border, intermediate and center of the open field. 7 to 10 mice of the indicated genotypes were recorded each for 10 min. (A) Activity in the open field and (B) duration of stay in the three indicated zones. αSYN^tg/tg^ G3Terc^-/-^ mice did not show a difference in comparison to αSYN^tg/tg^ mice and the two control groups. Although a trend can be seen in duration, αSYN^tg/tg^ and αSYN^tg/tg^ G3Terc^-/-^ stay even shorter in the intermediate and the center zone and prefer the border zone in comparison to Terc^+/+^ or G3Terc^-/-^ mice. (C) String agility test shows no difference between the different groups. (D) Activated astrocytes were measured using GFAP immunohistochemistry. The histogram depicts GFAP positive astrocytes of the brainstem (*n* = 4–6 mice). αSYN^tg/tg^ mice show a significant increase in astrocyte activation (62.35 ± 5.656) in comparison to αSYN^tg/tg^ G3Terc^-/-^ mice (39 ± 2.383, *P* = 0.0159). Furthermore, αSYN^tg/tg^ mice with phenotype (85 weeks) show a significant activation (62.35 ± 5.65) if compared with non-phenotypic mice (75 weeks, 33.37 ± 4.086, *P* = 0.0095). Terc^+/+^ (14.3 ± 7.6) and G3Terc^-/-^ mice (10.07 ± 7.10) show low activation levels. (PDF 33 kb) Additional file 5: Figure S4. Schematic representation of the parameter analyzed in 3D-reconstructed microglia. Automated analysis of each structural characteristic was performed using Imaris Bitplane software. (PDF 50 kb)
