# Supplementary material for: Telomere shortening leads to an acceleration of synucleinopathy and impaired microglia response in a genetic mouse model
Source: Acta Neuropathol Commun. 2016 Aug 22;4(1):87. doi: 10.1186/s40478-016-0364-x (PMC4994259; doi:10.1186/s40478-016-0364-x)
Supplement: Additional file 1: Table S2. — Primer sequences which were used for RT-PCR. (DOC 40 kb) [file 40478_2016_364_MOESM1_ESM.doc]

**Additional file 1: Table S2:** Primer sequences which were used for RT-PCR.

| **Primer Name** | **Sequence 5´ `3** |
| --- | --- |
| TNFα forward | 5´- CTGTAGCCCACGTCGTAGC |
| TNFα reverse | 5´- TTGAGATCCATGCCGTTG |
| IL 10 forward | 5´- CAGAGCCACATGCTCCTAGA |
| IL 10 reverse | 5´- TGTCCAGCTGGTCCTTTGTT |
| TGF-ß forward | 5´- TGGAGCTGGTGAAAGGAAG |
| TGF-ß reverse | 5´- ACAGGATCTGGCCACGGAT |
| CD14 forward | 5´- GCAGATGTGGAATTGTACGG |
| CD14 reverse | 5´- GTGTCCACACGCTTTAGAAGG |
| CD8 fororward | 5´- TGCTGTCCTTGATCATCACTCT |
| CD8 reverse | 5´ - ACTAGCGGCCTGGGACAT |
| Iba 1 forward | 5´- GGATTTGCAGGGAGGAAAAG |
| Iba 1 reverse | 5´- TGGGATCATCGAGGAATTIG |
| CD4 forward | 5´- TATCCAGAGGGGTGAACCAG |
| CD4 reverse | 5´- GAAACCCAGAAAGCCGAAG |
| llß forward | 5´- TGTAATGAAAGACGGCACACC |
| llß reverse | 5´- TCTTCTTTGGGTATTGCTTGG |
| Cxcl10 forward | 5´- GGGCCAGTGAGAATGAGGG |
| Cxcl10 forward | 5´- GCTCGCAGGGATGATTTCAA |
| CD80 forward | 5´- TTCGTCTTTCACAAGTGTCTTCA |
| CD80 reverse | 5´- TGCCAGTAGATTCGGTCTTCA |
| Cxcr1 forward | 5´- TGCAAGAATCGCAAGAAGG |
| Cxcr1 reverse | 5´- GAGGAAGAAGGCAAAGACCA |
| MHC- II forward | 5´- CACCGAGGCTCCACCTAA |
| MHC- II reverse | 5´- GCAGGGATGTGGCTGACT |
