# Supplementary figures and images for: Telomere shortening leads to an acceleration of synucleinopathy and impaired microglia response in a genetic mouse model
Source: Acta Neuropathol Commun. 2016 Aug 22;4(1):87. doi: 10.1186/s40478-016-0364-x (PMC4994259; doi:10.1186/s40478-016-0364-x)

Suppl. Fig. 1

A

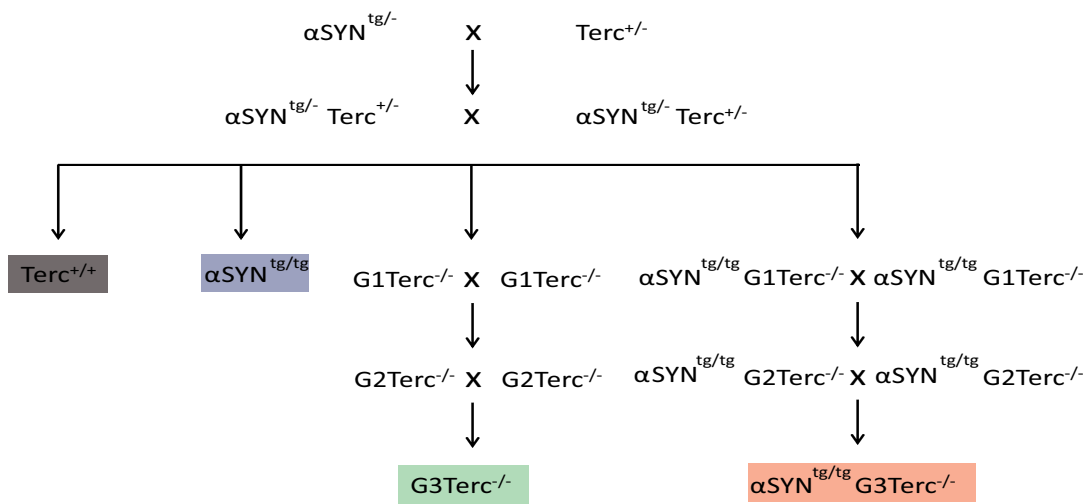

B

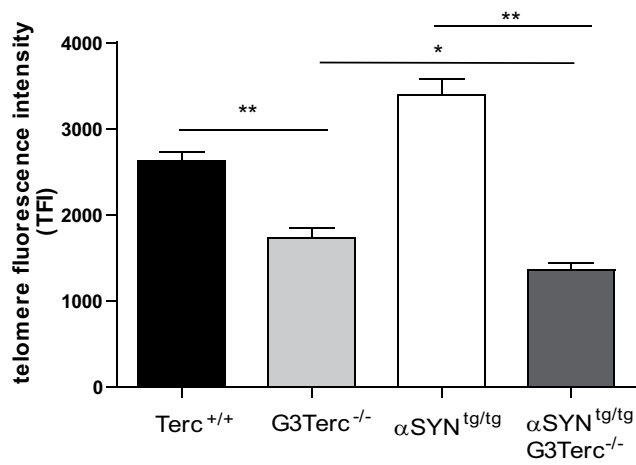

Supplement: Additional file 2: Figure S1. — Mating scheme for the generation of mouse cohorts. (A) Breeding scheme for generating 3rd generation telomerase knockout and α-synuclein transgenic mice (αSYNtg/tg G3Terc-/-) and corresponding control cohorts. Heterozygous α-synuclein (αSYN) mice were crossed with heterozygous telomerase knockout mice (Terc) to generate double transgenic αSYNtg/tg G1Terc-/- and single transgenic αSYNtg/tg, G1Terc-/- and Terc+/+ mice (G1 = first generation of telomerase knockout). These double transgenic αSYNtg/tg G1Terc-/- mice were crossed with each other to produce αSYNtg/tg G2Terc-/- mice and finally αSYNtg/tg G3Terc-/- mice. (B) Telomere length was measured in neurons of the brainstem using qFISH telomere staining double-stained with Cy5-Neuron N dye. The graph represents telomere length in brainstem in 75 weeks old mice (n = 5 mice per group). Analyzed were αSYNtg/tg G3Terc-/- in comparison to αSYNtg/tg mice (P = 0.0012) and G3Terc-/- in comparison to Terc+/+ mice (P = 0.0079) as well as αSYNtg/tg G3Terc-/- compared with G3Terc-/- (P = 0.03). (PDF 29 kb) [file 40478_2016_364_MOESM2_ESM.pdf]

Suppl. Fig. 2

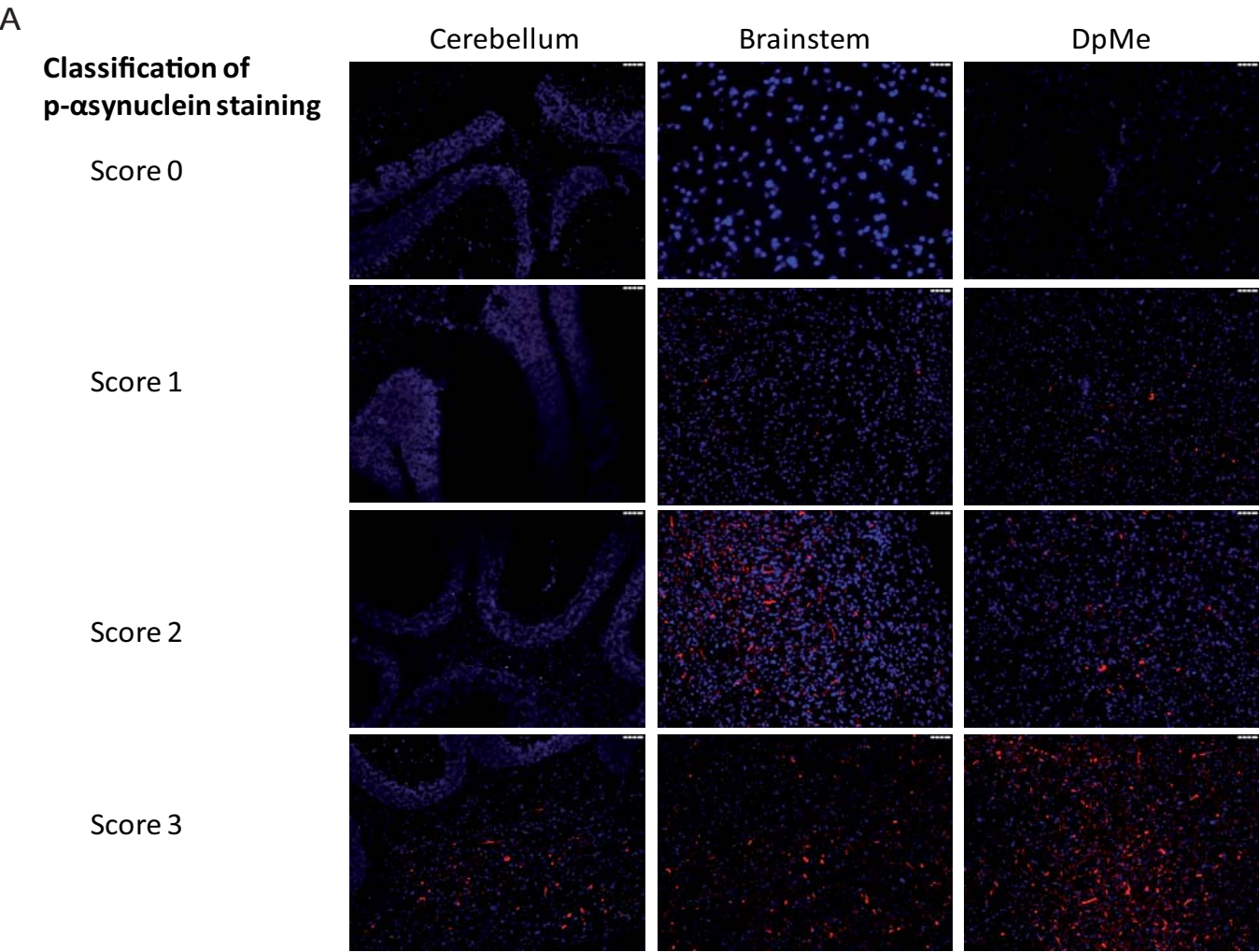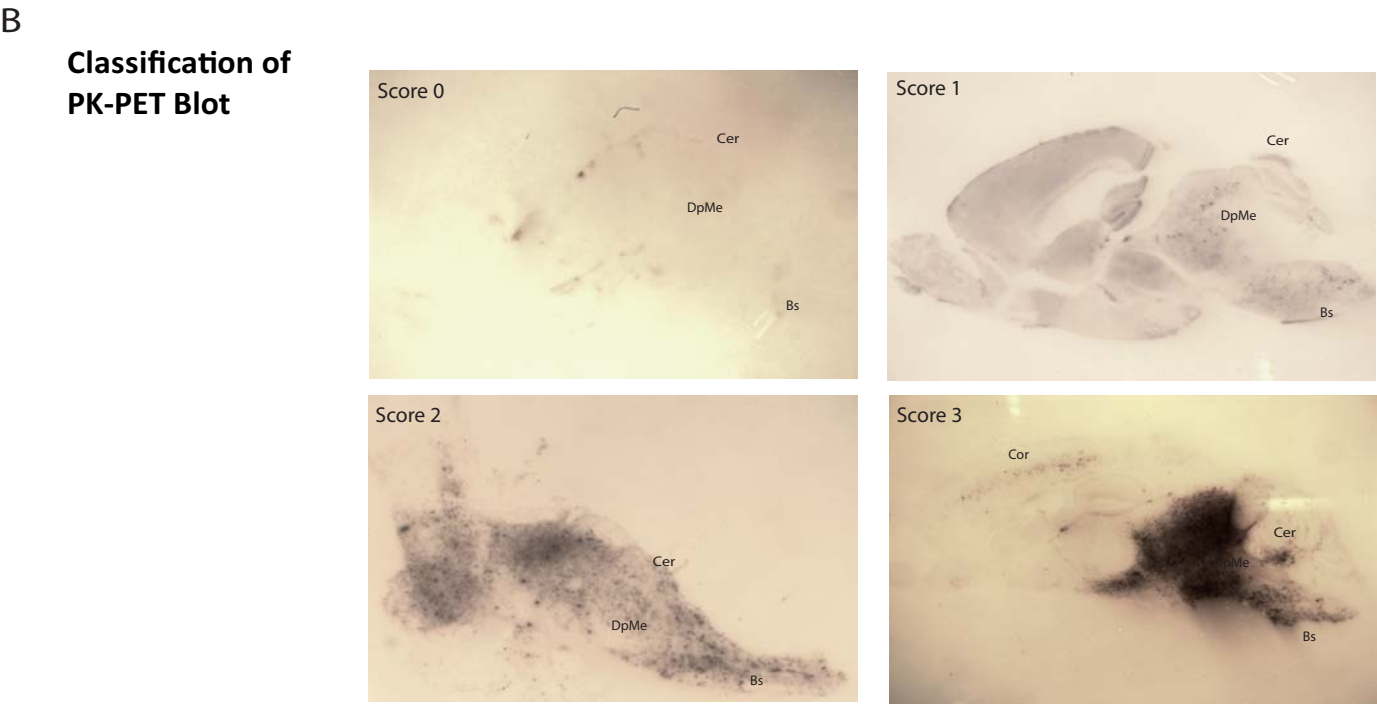

Supplement: Additional file 3: Figure S2. — Classification and scoring of phospho-α-synuclein and PK-PET Blot. (A) Classification of phospho-α-synuclein staining into four different scores. Representative pictures for scoring. Score 0: no p-α-synuclein staining, score 1: little staining in brainstem and DpMe, score 2: strong staining in brainstem and DpMe, score 3: strong p-α-synuclein staining in brainstem, DpMe, and cerebellum indicating severe disease progression. (B) Scoring to classify PK-PET Blot. Score 0: no PK resistant aggregates, score 1: light aggregates in brainstem and Deep Mesencepahlic nucleus (DpMe), score 2: clear PK resistant aggregates in brainstem and DpMe, score 3: dominant aggregates in brainstem and DpMe. Score 4: Prominent aggregates in brainstem, DpMe and cerebellum. (PDF 125 kb) [file 40478_2016_364_MOESM3_ESM.pdf]

Suppl. Fig. 3

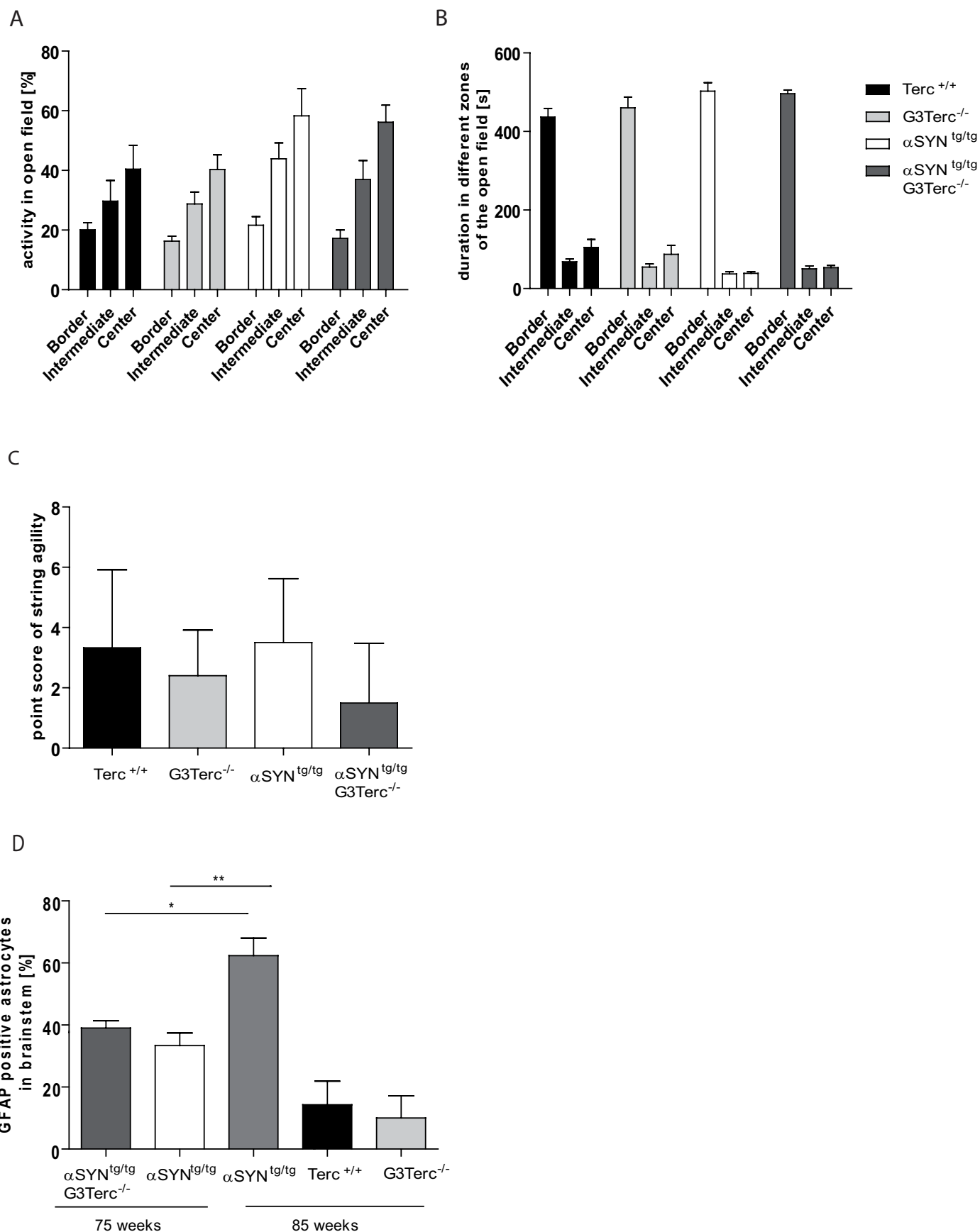

Supplement: Additional file 4: Figure S3. — Exploratory behavior and string agility. Exploratory behavior of the mice has been analysed using the Open Field System. Several parameters, e.g. the activity, duration and the totally travelled distance were measured in three different zones of the open field: Border, intermediate and center of the open field. 7 to 10 mice of the indicated genotypes were recorded each for 10 min. (A) Activity in the open field and (B) duration of stay in the three indicated zones. αSYNtg/tg G3Terc-/- mice did not show a difference in comparison to αSYNtg/tg mice and the two control groups. Although a trend can be seen in duration, αSYNtg/tg and αSYNtg/tg G3Terc-/- stay even shorter in the intermediate and the center zone and prefer the border zone in comparison to Terc+/+ or G3Terc-/- mice. (C) String agility test shows no difference between the different groups. (D) Activated astrocytes were measured using GFAP immunohistochemistry. The histogram depicts GFAP positive astrocytes of the brainstem (n = 4–6 mice). αSYNtg/tg mice show a significant increase in astrocyte activation (62.35 ± 5.656) in comparison to αSYNtg/tg G3Terc-/- mice (39 ± 2.383, P = 0.0159). Furthermore, αSYNtg/tg mice with phenotype (85 weeks) show a significant activation (62.35 ± 5.65) if compared with non-phenotypic mice (75 weeks, 33.37 ± 4.086, P = 0.0095). Terc+/+ (14.3 ± 7.6) and G3Terc-/- mice (10.07 ± 7.10) show low activation levels. (PDF 33 kb) [file 40478_2016_364_MOESM4_ESM.pdf]
