# Supplementary material for: Telomere shortening leads to an acceleration of synucleinopathy and impaired microglia response in a genetic mouse model
Source: Acta Neuropathol Commun. 2016 Aug 22;4(1):87. doi: 10.1186/s40478-016-0364-x (PMC4994259; doi:10.1186/s40478-016-0364-x)
Supplement: Additional file 5: Figure S4. — Schematic representation of the parameter analyzed in 3D-reconstructed microglia. Automated analysis of each structural characteristic was performed using Imaris Bitplane software. (PDF 50 kb) [file 40478_2016_364_MOESM5_ESM.pdf]

Suppl. Fig. 4

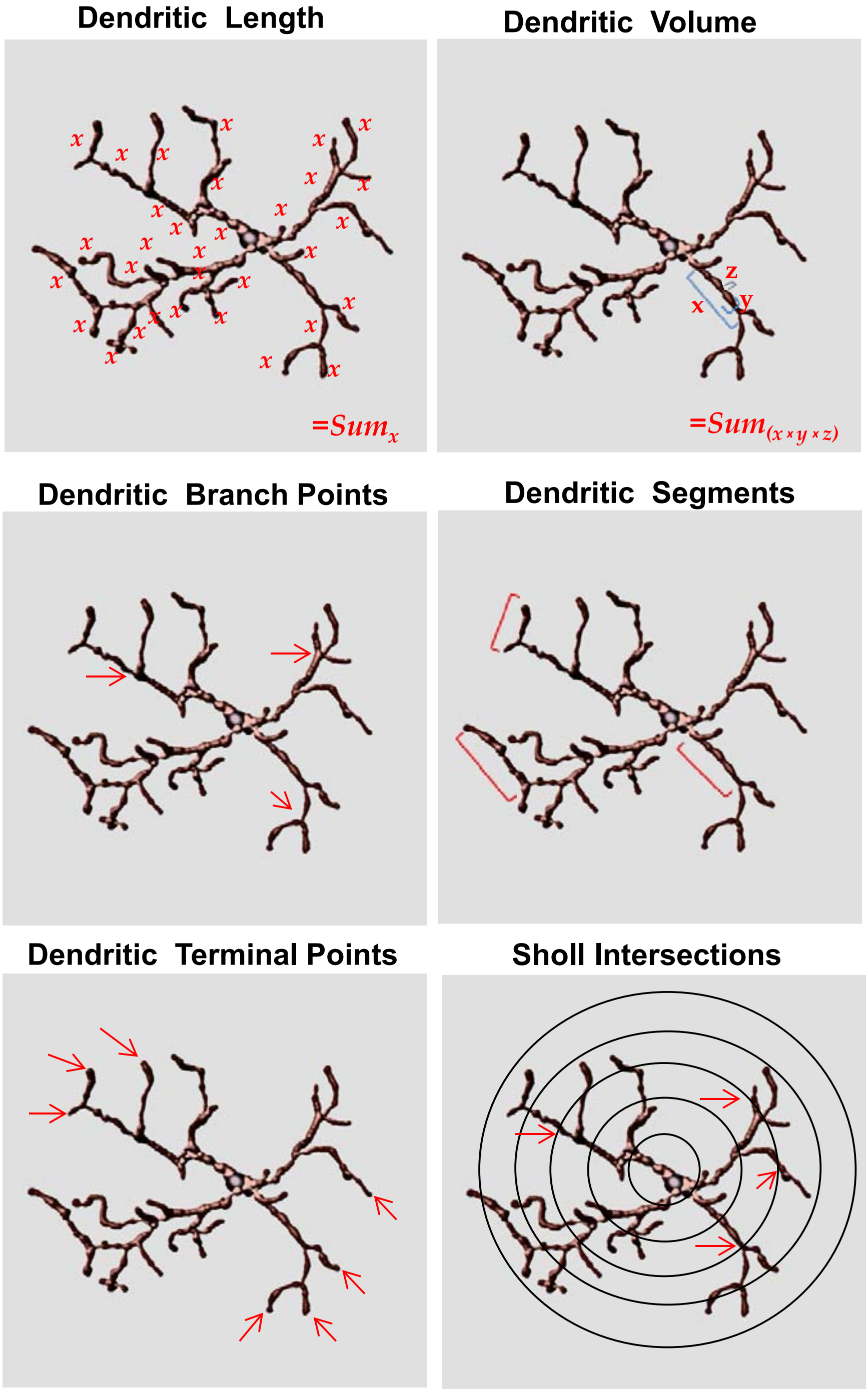

Dendritic Length
=Sum
Dendritic Volume
z
y
x
=Sum
Dendritic Branch Points
Dendritic Segments
Dendritic Terminal Points
Sholl Intersections
